# Development and evaluation of a chronic kidney disease risk prediction model using random forest

**DOI:** 10.3389/fgene.2024.1409755

**Published:** 2024-06-27

**Authors:** Krish Mendapara

**Affiliations:** Faculty of Health Sciences, Queen's University, Kingston, ON, Canada

**Keywords:** CKD, chronic kidney disease, random forest., biomarkers, computational genomics and proteomics, disease risk prediction algorithm, differentially expressed genes (DEGs)

## Abstract

This research aims to advance the detection of Chronic Kidney Disease (CKD) through a novel gene-based predictive model, leveraging recent breakthroughs in gene sequencing. We sourced and merged gene expression profiles of CKD-affected renal tissues from the Gene Expression Omnibus (GEO) database, classifying them into two sets for training and validation in a 7:3 ratio. The training set included 141 CKD and 33 non-CKD specimens, while the validation set had 60 and 14, respectively. The disease risk prediction model was constructed using the training dataset, while the validation dataset confirmed the model’s identification capabilities. The development of our predictive model began with evaluating differentially expressed genes (DEGs) between the two groups. We isolated six genes using Lasso and random forest (RF) methods—DUSP1, GADD45B, IFI44L, IFI30, ATF3, and LYZ—which are critical in differentiating CKD from non-CKD tissues. We refined our random forest (RF) model through 10-fold cross-validation, repeated five times, to optimize the mtry parameter. The performance of our model was robust, with an average AUC of 0.979 across the folds, translating to a 91.18% accuracy. Validation tests further confirmed its efficacy, with a 94.59% accuracy and an AUC of 0.990. External validation using dataset GSE180394 yielded an AUC of 0.913, 89.83% accuracy, and a sensitivity rate of 0.889, underscoring the model’s reliability. In summary, the study identified critical genetic biomarkers and successfully developed a novel disease risk prediction model for CKD. This model can serve as a valuable tool for CKD disease risk assessment and contribute significantly to CKD identification.

## Highlights

1. Integration of various GEO datasets into a comprehensive sample dataset.
2. Examination of biomarkers for CKD within kidney tissue specimens.
3. A collaborative investigation utilizing Lasso and random forest methods to identify CKD biomarkers.
4. An innovative and robust CKD disease risk prediction model was created, employing a random forest algorithm and utilizing six critical genes (DUSP1, GADD45B, IFI44L, IFI30, ATF3, and LYZ).
5. Thorough testing of the models using both a validation dataset and an external validation dataset.

## Introduction

Chronic Kidney Disease (CKD) is defined by a progressive deterioration of renal function sustained over 3 months or more, independent of its underlying cause, eventually necessitating renal replacement therapy, such as dialysis or transplantation (Vaidya and Aeddula, 2023) Kidney damage encompasses pathological irregularities, which may be indicated by imaging investigations, renal biopsy, anomalies in urinary sediment, or elevated urinary albumin excretion rates (Vaidya and Aeddula, 2023). The diversity in clinical manifestations of CKD can impact various facets of bodily function, including the cardiovascular system, electrolyte equilibrium, bone health, anemia status, and overall metabolic wellbeing (Management of Progression, 2013; Dhondup and Qian, 2017; Gallant and Spiegel, 2017; Hutcheson and Goettsch, 2023). The disruption of multiple physiological systems plays a central role in the development of CKD. Considering that CKD encompasses a wide range of kidney diseases, its origins can span from glomerular damage, tubular dysfunction, and interstitial injury, to vascular impairment. Prominent factors such as diabetes, hypertension, and inflammation often play a substantial role in advancing and establishing chronic kidney conditions (Vaidya and Aeddula, 2023). CKD is an irreversible condition marked by a continual decline in kidney health, necessitating ongoing medication management for its symptoms (Mayo Clinic, 2023a). While the underlying mechanisms of its onset remain unclear, precise detection of CKD is crucial as it can significantly improve patient outcomes. Given the pivotal role of autoimmune dysregulation in CKD, immune biomarkers have surfaced as valuable tools for enhancing CKD diagnosis and, consequently, enhancing disease management (Espi et al., 2020). Nonetheless, the diverse and often ambiguous symptoms associated with CKD pose challenges in achieving an accurate and prompt diagnosis (Mayo Clinic, 2023b). The need for enhanced diagnostic and treatment strategies for CKD is immediate. Over the last 10 years, advancements in microarray sequencing have provided a reliable and thorough approach for deciphering the genetic and epigenetic factors of various diseases. Such a plethora of data also facilitates the prediction of a multitude of diseases (Gunn and Smith, 2004; Wang J. et al., 2022). Wang et al. demonstrated that utilizing multiple biomarkers in prediction models can notably enhance predictive accuracy (Wang et al., 2020). Selecting the right features remains a substantial obstacle in the creation of classification models based on multiple genes. Fortunately, this problem is being adeptly addressed by employing a range of machine-learning strategies in modern biological research (Kursa, 2014; Degenhardt et al., 2019). These algorithms, whether used in isolation or in combination, have made substantial contributions to gene expression data classification, disease detection, and microbiome studies (Liu et al., 2018; Hernández Medina et al., 2022; Alshammri et al., 2023).

We established a novel disease risk prediction model for CKD utilizing transcriptome data, focusing on the critical genes identified within the GEO database. Initially, we employed Lasso and RF techniques to identify influential genes for CKD classification. Subsequently, through grid search, we selected the optimal “mtry” parameter for the RF model, leading to the development of a genetic disease risk prediction model for CKD based on these key genes. We assessed the model’s performance using a validation dataset to validate its accuracy and discriminative capabilities.

## Materials and methods

### Data sources

The data utilized in this study were sourced from the Gene Expression Omnibus (GEO) database, an established repository for gene expression information managed by the National Center for Biotechnology Information (NCBI) (https://www.ncbi.nlm.nih.gov/geo/query/acc.cgi). To conduct the study, we conducted an extensive search within the NCBI database platform using the keyword “chronic kidney disease.” The selected dataset fell under the category of array expression profiling, featured the organism *Homo sapiens*, and comprised kidney tissue samples.

The datasets GSE35488, GSE32591, GSE66494 and GSE47184 were obtained. GSE35488 is a dataset containing 25 CKD patient samples and six healthy samples. The kidney tissue samples were obtained from the University of Michigan. The gene expression was analyzed using the Affymetrix GeneChip Human Genome HG-U133A Array (using custom CDF). GSE32591 is a dataset containing 64 CKD patient samples and 29 healthy samples. The kidney tissue samples were obtained from the University of Michigan. The gene expression was analyzed using the Affymetrix GeneChip Human Genome HG-U133A Array (using custom CDF). GSE66494 is a dataset containing 53 CKD patient samples and eight healthy samples. The kidney tissue samples were obtained from Kyushu University Hospital. The gene expression was analyzed using the Agilent-014850 Whole Human Genome Microarray 4 × 44K G4112F (Probe Name version). GSE47184 is a dataset containing 60 CKD patient samples and four healthy samples. The kidney tissue samples were obtained from the University of Michigan. Gene expression was analyzed using the Affymetrix GeneChip Human Genome HG-U133A (using custom CDF). GSE180394 (external dataset) is a dataset containing 50 CKD patient samples and nine healthy samples. The kidney tissue samples were obtained from the University of Michigan. Gene expression was analyzed using the Affymetrix Human Gene 2.1 ST Array. The information about the four datasets and the external validation dataset (GSE180394) is displayed in Table 1; Supplementary Table S1.

**TABLE 1:** The data regarding the gene expression datasets related to Chronic Kidney Disease (CKD) within the Gene Expression Omnibus (GEO) repository.

GEO accession	Expression profiling	Tissue	CKD	Control	Total
GSE35488	Array	Kidney tissue	25	6	31
GSE32591	Array	Kidney tissue	64	29	93
GSE66494	Array	Kidney tissue	53	8	61
GSE47184	Array	Kidney tissue	60	4	64
GSE180394	Array	Kidney tissue	50	9	59

These datasets were chosen for their breadth of chronic kidney disease (CKD) stages and types, aiming to develop a model that generalizes CKD across these stages. GSE35488 contains primary glomerulonephritis and IgA nephropathy, covering both early and late stages. GSE32591 contains lupus nephritis, covering both early and late stages. GSE66494 contains chronic kidney disease, covering both early and late stages. GSE47184 contains diabetic nephropathy, minimal change disease, thin membrane disease, focal and segmental glomerulosclerosis, hypertensive nephropathy, IgA nephropathy, membranous glomerulonephritis, and rapidly progressive glomerulonephritis, covering both early and late stages. GSE180394 contains focal and segmental glomerulosclerosis, chronic glomerulonephritis, diabetic nephropathy, IgA nephropathy, interstitial nephritis, hypertensive nephrosclerosis, light-chain deposit disease, lupus nephritis (various WHO classes), minimal change disease, membranous nephropathy, and chronic kidney disease with moderate to severe interstitial fibrosis, covering both early and late stages.

### Data processing

Next, we initiated a sequence of data processing procedures on our merged gene expression dataset to ensure accuracy and consistency. To begin, we implemented quantile normalization across the entire expression dataset, ensuring a consistent distribution of probe intensities. Following this, we introduced a log2 transformation to improve the interpretability and comparability of expression values. Finally, to address and rectify any potential batch effects that could arise from differences in experimental platforms, we employed the ComBat function from the sva package. This method efficiently harmonized the data, mitigating discrepancies between the batches (Supplementary Figure S1). By removing noise and potential biases in the data, this process enhanced the model’s generalization ability and ensured more reliable predictions across different experimental conditions.

### Stratified random sampling

To enhance the model’s ability to accurately reflect its robustness in disease risk prediction, we employed a stratified random sampling approach to ensure an equitable sample distribution. Utilizing the “createDataPartition” function from the R package caret, we partitioned the array expression spectrum datasets (GSE35488, GSE32591, GSE66494, and GSE47184) into both a training dataset and a validation dataset, maintaining a sample size ratio of 7:3. Subsequently, we utilized the training dataset for the development of the disease risk prediction model, while the validation dataset was employed to assess the model’s effectiveness.

### Screening for DEGs

We conducted differential expression analysis on the training dataset to identify Differentially Expressed Genes (DEGs) using conventional Bayesian approaches via the limma package. Significance criteria for DEGs were established with a false discovery rate (FDR) threshold <0.05 and an absolute log2 fold change (log2FC) > 1. Subsequently, we generated a heatmap of the DEGs utilizing the pheatmap package and created a volcano map using the ggplot2 package.

### Gene enrichment analysis

The analysis of Differentially Expressed Genes (DEGs) was undertaken with the application of Gene Ontology (GO), Kyoto Encyclopedia of Genes and Genomes (KEGG), and Disease Ontology (DO). Specifically, for GO and KEGG analysis, we employed the clusterProfiler package, and for DO analysis, we utilized the DOSE package. The interpretation of Gene Ontology primarily focused on biological processes (BP). This analysis allows us to gain valuable insights into the biological functions and pathways associated with the DEGs.

### Feature selection

We conducted Lasso regression using the glmnet package as part of our feature selection process. Lasso regression is a robust feature selection algorithm known for effectively addressing collinearity issues and identifying representative variables. By constraining the sum of the absolute values of the model parameters, Lasso helps in shrinking some coefficients to zero, thereby facilitating the selection of a simpler, more interpretable model. This technique is particularly advantageous in high-dimensional datasets, where multicollinearity can be a significant issue. Lasso regression’s ability to penalize the coefficients and minimize overfitting makes it a suitable choice for identifying key biomarkers in our study. To further refine our feature selection, we employed the recursive feature elimination (RFE) method in conjunction with a random forest classifier, a step facilitated by the caret package. RFE iteratively removes the least important features based on the model’s performance, thus narrowing down the feature set to the most impactful variables. This approach enhances the model’s interpretability and robustness by ensuring that only the most relevant features are retained. Additionally, RFE’s integration with a random forest classifier leverages the inherent feature importance metrics of the ensemble method, providing a comprehensive assessment of each feature’s contribution to the predictive model. We complemented this process with a 10-fold cross-validation to ensure the reliability and validity of our feature selection. Cross-validation involves partitioning the data into ten subsets, training the model on nine subsets, and validating it on the remaining subset, repeating this process ten times. This technique helps in mitigating overfitting and provides a more accurate estimation of the model’s performance on unseen data. By combining RFE with cross-validation, we ensure a robust feature selection process that balances model complexity and predictive accuracy, ultimately enhancing the overall efficacy of our chronic kidney disease risk prediction model.

Subsequently, we employed a grid search to pinpoint the optimal mtry parameter for fitting the random forest dataset. This was followed by a process of 10-fold cross-validation, which included five iterations and several rounds of training. Using the caret and randomForest packages, we then proceeded to evaluate the significance scores of the feature genes. In our final step, we focused on the signature genes, identifying those with importance scores >25 as the most important.

It is important to emphasize that the mtry parameter, representing the count of variables randomly sampled when building decision tree branches in random forest models, is crucial. Its appropriate selection is key to minimizing prediction errors and boosting the overall performance of the model.

### Constructing random forest predictive model for CKD

In our CKD disease risk prediction model, we incorporated the chosen signature genes using a random forest model. Random forest, an ensemble learning method, is highly effective for classification tasks due to its robustness and ability to handle large datasets with higher dimensionality. By constructing multiple decision trees during training and outputting the mode of the classes (classification) or mean prediction (regression) of the individual trees, random forest mitigates overfitting and improves predictive accuracy. The integration of random forest in our model ensures that we leverage its strengths in capturing complex interactions between features and enhancing the overall stability and performance of the prediction model.

To achieve an optimally fitted random forest dataset, we applied a grid search technique, using the caret and randomForest packages, to determine the best mtry parameter. This was followed by a 10-fold cross-validation procedure, involving five rounds of repetition and numerous training cycles. The primary goal of this process was to improve the model’s performance and reduce the risk of overfitting, with a focus on evaluating accuracy metrics. Ultimately, we constructed the CKD random forest diagnostic model, equipped with the optimal mtry parameters. To determine its robustness, we subjected this model to 5-fold cross-validation on the training dataset and assessed the accuracy of the results using the confusionMatrix function. Additionally, we computed the area under the receiver operator characteristic (ROC) curve (AUC) using the pROC package to gauge the model’s discriminatory capability.

### Verification using validation datasets

The validation dataset, as well as the external validation dataset (GSE180394), provided robust confirmation of the efficacy of our CKD disease risk prediction model built through random forest. To account for potential batch effects between GSE180394 (Supplementary Figure S2) and the training dataset, we once again applied the Combat function for adjustment before conducting model testing.

To further validate the reliability of our biomarkers, we ascertained the optimal parameter mtry, assessed the area under the curve (AUC), and evaluated the accuracy of our optimal disease risk prediction model for CKD using random forest. The AUC was computed using the pROC package, while accuracy measurements were obtained through the confusionMatrix function.

### Statistical analysis

We conducted all statistical analyses using R software (version 4.3.1), and statistical significance was defined as *p* < 0.05.

## Results

### Study design

**FIGURE 1:**
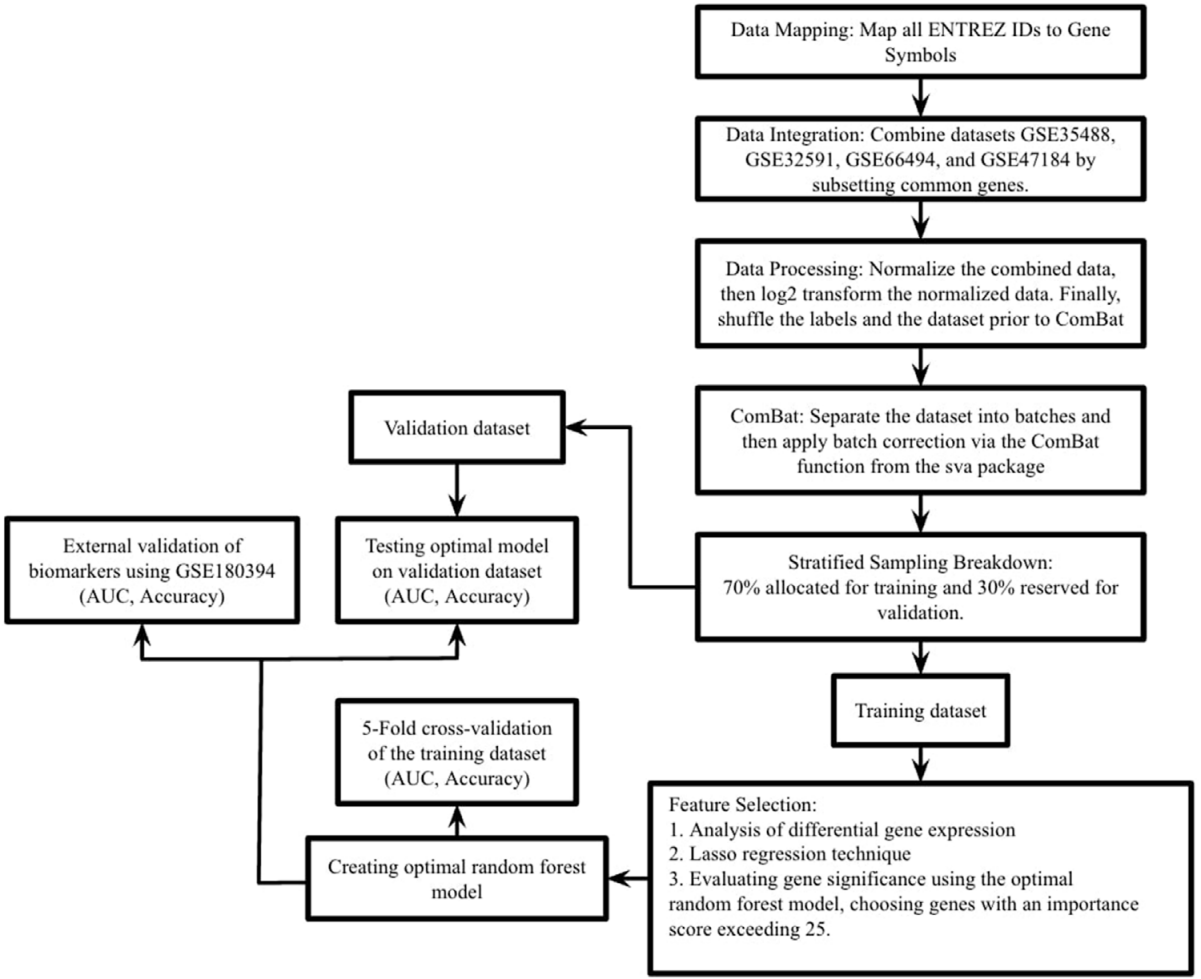
This study’s process is outlined as follows. In the first step, datasets GSE35488, GSE32591, GSE66494, and GSE47184 were merged into a single comprehensive dataset. The second step involved dividing this extensive dataset into training and validation sets using stratified random sampling, adhering to a 7:3 ratio. The third step focused on the training dataset, where differential expression analysis was performed, Lasso regression and RF-RFE (Random Forest - Recursive Feature Elimination) were executed, and the feature importance score of RF was used to pinpoint essential genes. In the fourth step, these key genes were integrated into a random forest prediction model. The fifth step entailed evaluating the model’s effectiveness through 5-fold cross-validation on the training set. Additionally, the model’s robustness was tested using the validation set and an external validation dataset (GSE180394), with performance measured in terms of the area under the curve (AUC), accuracy, and sensitivity.

Figure 1 illustrates the complete study process.

### Identification of DEGs

**FIGURE 2:**
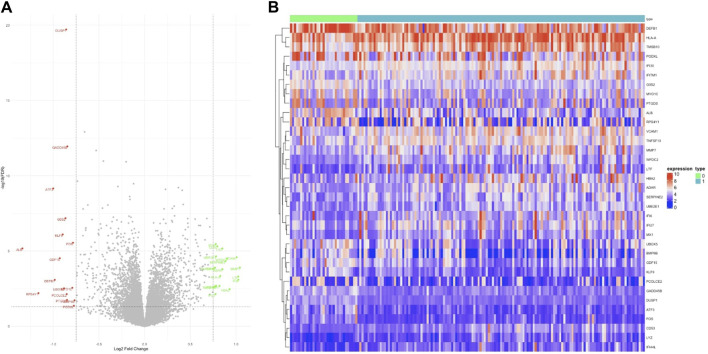
Differentially expressed genes. **(A)** In the volcano plot, 35 genes are highlighted for their significant differential expression, with green dots indicating upregulated genes, black dots for genes with no notable differences, and red dots for downregulated genes. **(B)** The heat map illustrates the expression patterns of these 35 genes, clearly showing trends of both upregulation and downregulation.

We employed a stratified random sampling method to partition the data into a training dataset (70%) and a validation dataset (30%). In the training dataset, there were 141 CKD samples and 33 healthy samples, while the validation dataset comprised 60 CKD samples and 14 healthy samples. Subsequently, we conducted differential expression analysis on the training dataset to identify differentially expressed genes (DEGs) associated with CKD. We identified a total of 35 significant DEGs based on predefined significance criteria. To visualize the expression patterns of all DEGs, we generated a volcano plot (Figure 2A), which revealed a nearly equal distribution of upregulated and downregulated genes. The heat map analysis (Figure 2B) further illustrated significant differences in the expression levels of DEGs between the CKD (designated as ‘1′) and control group (designated as ‘0′).

### Enrichment analysis

**FIGURE 3:**
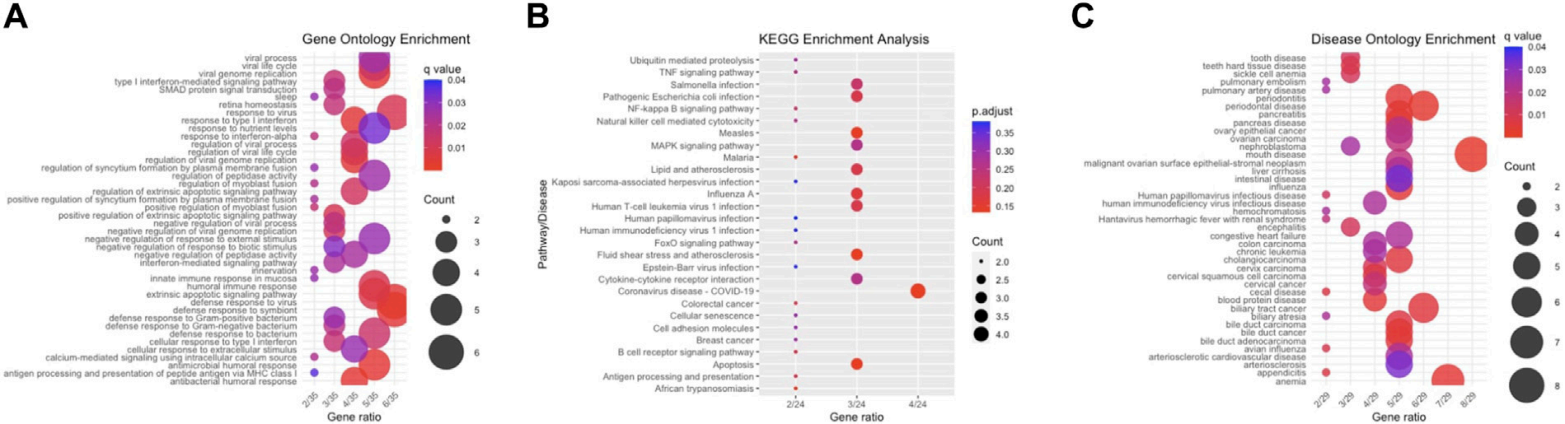
Enrichment Analysis. **(A)** This section features a bar plot representing biological processes derived from GO enrichment analysis. **(B)** It includes a bar plot depicting the results of KEGG enrichment analysis. **(C)** The section concludes with a bar plot showing the findings from DO enrichment analysis.

We conducted enrichment analyses for the 35 differentially expressed genes (DEGs), including Gene Ontology (GO), Kyoto Encyclopedia of Genes and Genomes (KEGG), and Disease Ontology (DO) analyses. Regarding biological processes (Figure 3A), the results revealed significant enrichment of DEGs in response to viral and immune responses. In the context of KEGG analysis (Figure 3B), our findings indicated prominent enrichment in pathways related to viral-associated diseases, specifically those involving MAPK, NF-κB, and IL-6, along with cytokine receptor signalling pathways. As for DO analysis (Figure 3C), it suggested a close association between crucial genes linked to CKD and autoimmune diseases such as intestinal disease, mouth disease, and atherosclerosis.

### Key gene selection

**FIGURE 4:**
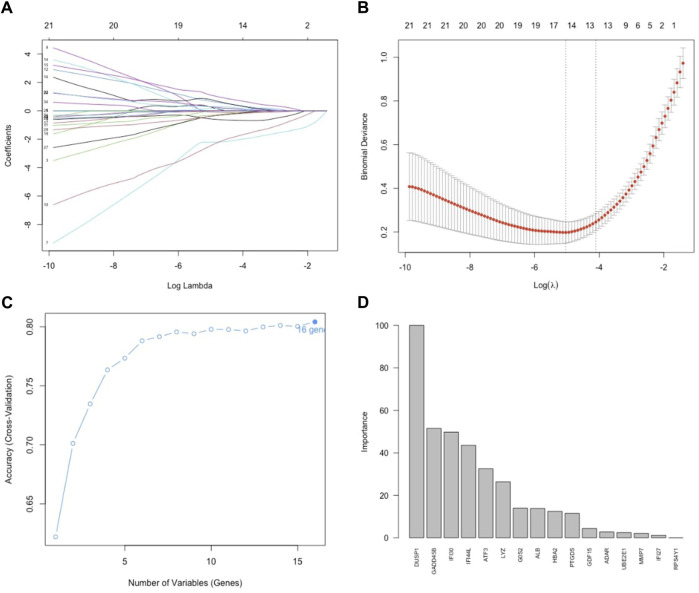
Feature selection. **(A)** Lasso regression curve depicting the 35 DEGs. **(B)** Options for the λ parameter in the 10-fold cross-validation. **(C)** RMSE values for the 10-fold cross-validation of the RF-RFE-selected signature gene combination. **(D)** Importance scores of genes in the random forests model. Development of the random forest model.

To identify key genes, we first employed Lasso regression with a 10-fold cross-validation on the initial set of 35 DEGs. Using Lambda as the criterion (Figures 4A, B), we selected 16 candidate genes by reducing the feature variables. We proceeded with feature selection using RF-RFE, as shown in Figure 4C. This process revealed that incorporating all 16 candidate genes resulted in the highest accuracy for the model. In the concluding phase, we refined the model by incorporating these 16 genes into a random forest classifier and performing 10-fold cross-validation five times. To ensure robust predictive capability while minimizing the number of features, we focused on six genes with importance scores above 25, designating them as the key genes. Figure 4D displays the significance of these genes, with DUSP1 being the most pivotal, followed by GADD45B, IFI30, IFI44L, ATF3, and LYZ.

### Development of the random forest model

**FIGURE 5:**
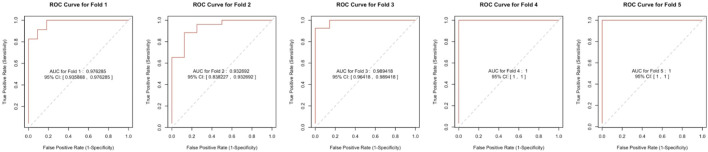
The ROC curve results were confirmed by a 5-fold cross-validation.

**FIGURE 6:**
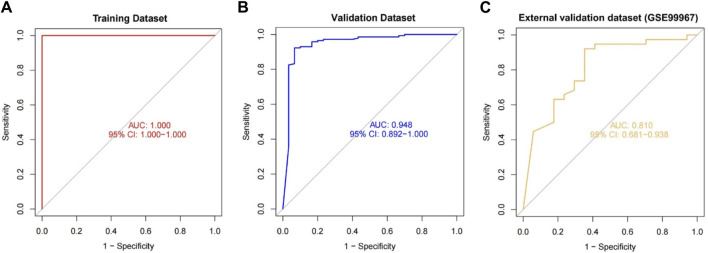
The performance of the random forest model was evaluated across the training **(A)**, validation **(B)**, and external validation **(C)** datasets, utilizing ROC curves and analyzing their respective AUC values.

We included DUSP1, GADD45B, IFI30, IFI44L, ATF3, and LYZ in the random forest classifier. To enhance model performance, we conducted a grid search for the mtry parameters and assessed model accuracy for each mtry through 10-fold cross-validation repeated 5 times. Subsequently, we established the optimal random forest disease risk prediction model with an mtry of 2. We then conducted a robustness assessment using a 5-fold cross-validation, representing results with ROC curves (Figure 5), and presenting accuracy values in Table 2. The average AUC from the 10-fold cross-validation results exceeded 0.97, confirming the model’s reliability. Finally, we evaluated the AUC and accuracy for the entire training dataset, resulting in an AUC of 1% and 100% accuracy (Figure 6A).

**TABLE 2:** The 5-fold cross-validation results.

	Accuracy (%)	AUC
Cross-Validation Fold 1	88.235	0.976
Cross-Validation Fold 2	88.235	0.933
Cross-Validation Fold 3	94.118	0.989
Cross-Validation Fold 4	94.118	1.000
Cross-Validation Fold 5	91.176	1.000

### Random forest validation

In the validation dataset, the ROC curve analysis yielded an AUC value of 0.990, and the confusion matrix estimated an accuracy of 94.595%. These results underscore the model’s robustness in identifying CKD (Figure 6B). This demonstrates the successful development of a CKD disease risk prediction model based on differential gene expression between CKD and normal samples. Moreover, we constructed models with and without DUSP1, resulting in respective AUCs of 0.880 and 1.00 (Supplementary Figure S3). Interestingly, adding the most crucial gene, DUSP1, enhanced rather than diminished model performance, as evident from the AUC values in the previous validation set of the six-gene disease risk prediction model. In addition, on the validation dataset, the model achieved a precision of 0.916, a recall of 0.786, and an F1 score of 0.846.

### Random forest model validation using external dataset

For additional validation, we applied our model to an external dataset (GSE180394). Analysis of the ROC curve from this dataset resulted in an AUC of 0.913, as shown in Figure 6C, reflecting the model’s robust diagnostic discrimination. Through the confusion matrix, we calculated an accuracy rate of 89.83% and a sensitivity of 0.889. This sensitivity rate is particularly significant as it underscores the model’s enhanced ability to accurately identify CKD in individuals.

## Discussion

Chronic Kidney Disease (CKD) is a medical condition marked by the progressive and irreversible deterioration of renal function, stemming from the gradual breakdown of kidney tissue (PACE Hospital, 2023). Accurate prediction and early detection play a pivotal role in enhancing the survival rates of individuals with CKD (Shlipak et al., 2021). Despite ongoing research, the exact process of CKD’s development is not fully understood. At present, standard diagnostic methods for CKD rely primarily on serum creatinine tests, which include assessments of Glomerular Filtration Rate (GFR) and urine Albumin-to-Creatinine Ratio (ACR) (Chen et al., 2019). Clinical diagnosis of CKD is rarely made promptly because of its relatively slow-developing nature (Rosenberg, 2021), CKD patients are often asymptomatic, and definitive confirmation typically requires specific laboratory tests and monitoring over an extended period to establish a consistent pattern of kidney dysfunction (Chen et al., 2019). It is essential to identify biomarkers that have a strong correlation with CKD. Thanks to machine learning advancements and publicly available gene expression data, we can now more effectively identify biomarkers strongly linked to diseases (Aromolaran et al., 2021).

In our research, we developed a CKD disease risk prediction model using the random forest algorithm to distinguish between CKD patient renal tissue and normal renal tissue. With the rapid advancement of bioinformatics, we now have strong evidence to support disease classification like CKD. To identify CKD’s Differentially Expressed Genes (DEGs), we integrated data from four GEO datasets (GSE35488, GSE32591, GSE66494, and GSE47184) and utilized stratified random sampling to divide the dataset into training (70%) and validation (30%) sets. Following this, we carried out enrichment analyses using GO, KEGG, and DO, which showed that the differentially expressed genes (DEGs) are linked to a diverse range of biological processes and pathways. This indicates the complexity of the underlying mechanisms in CKD pathogenesis. Numerous research efforts align with our results, and prior studies have demonstrated MAPK, NF-κB, Interleukin-6 (IL-6) and cytokine-receptor pathways are key in the pathogenesis of CKD (Bruggeman, 2007). A recent study by Kristen Kurtzeborn et al. (Kurtzeborn et al., 2019) explored the role of MAPK/ERK signalling in renal differentiation, shedding light on the pathway’s involvement in nephrogenesis and its relevance to kidney degradation. Yuan et al. (Yuan et al., 2022) identified MAPK as a key signalling pathway linked to chronic kidney disease (CKD), contributing to lipotoxicity and oxidative stress. Inflammatory pathways like NF-κB, and Cytokine receptor signaling were also identified as central to CKD progression. In their study, Su et al. (Su et al., 2017) highlighted kidney resident cells, specifically podocytes secrete IL-6 under certain conditions to promote proliferation; affecting the differentiation of kidney cells. The concentration of various inflammatory signalling pathways and diseases related to inflammation in CKD patients suggests a link between CKD pathogenesis and autoimmune irregularities, a viewpoint widely accepted among CKD researchers (Berthier et al, 2012; Chen et al., 2023). Among them, viral infections, lipids, and atherosclerosis were positively correlated with CKD, which suggests that CKD patients are prone to cardiovascular and autoimmune diseases (Gorenjak, 2009; Olechnowicz-Tietz et al., 2013), and the development of such diseases is closely related to immune responses. We found that necrosis is upregulated in CKD patients and that necrosis is closely associated with damage-associated molecular patterns (DAMPs), resulting in an excessive immune reaction (Green et al., 2009; Sarhan et al., 2018). Notably, pathways involving MAPK, IL-6, and NF-kB have been shown to play significant roles in immune responses. For instance, soluble CRT can activate the MAPK and NF-κB pathways, leading to the production of pro-inflammatory cytokines like TNF-α and IL-6 in macrophages (Montico et al., 2018). Furthermore, NF-κB, known for its pro-survival transcriptional activity, can upregulate antiapoptotic genes, contributing to cell survival (Verzella et al., 2020), thereby promoting a heightened immune response as a defence mechanism against tumour growth. Additionally, during a typical immune response, dendritic cells (DCs) capture antigens and release cytokines, including IL-6, that shape immune cell responses, such as those of Natural Killer cells (NK) and T cells, which receive survival signals and stimulation through cytokines like IL-6 (Showalter et al., 2017). It has also been recently proposed that the process of necroptosis might play a role in the pathogenesis and progression of CKD and that elevated IL-6/NF-κB/MAPK signalling in CKD increases necroptosis, which leads to tissue damage (Tanaka et al., 2014). However, the study of necroptosis in CKD is still poorly studied, and its contribution to the disease’s pathogenesis and progression requires more in-depth investigation.

Further performance of the RF classifier importance score screened for six key genes, namely, DUSP1, GADD45B, IFI30, IFI44L, ATF3, and LYZ. Previous studies support our findings. Dual-Specificity Phosphatase 1 (DUSP1), one of the enzymes for mitogen-activated protein kinases (MAPKs), plays a key role of initiating MAPK cascade (Li et al., 2021). Growth Arrest and DNA Damage Inducible Beta (GADD45B) is also often implicated in the pathogenesis of CKD (Moon et al., 2020). GADD45B serves as an anti-apoptotic factor, exerting its effects by directly binding to MKK7. Through this interaction, GADD45B effectively suppresses the MKK7-dependent phosphorylation of JNK1/2, thus preventing apoptosis (Yamamoto et al., 2010). Gamma-interferon lysosomal thiol reductase (IFI30) is an enzyme that is expressed constitutively in antigen-presenting cells (NCBI, 2024a), having been shown to reduce protein disulfide bonds in endocytosis-mediated protein degradation. An IFI30 deficiency impaired endothelial cells and macrophages, including cell proliferation and migration (Wang X. et al., 2022). Interferon-induced Protein 44 Like (IFI44L) acts as a feedback regulator of IFN responses (DeDiego et al., 2019), which are a group of signalling proteins secreted by host cells in reaction to the presence of multiple viruses (Murira and Lamarre, 2016). Functional enrichment analysis has revealed that IFI44L might be involved in numerous immune-related pathways, including inhibiting the NF-κB signalling pathway (DeDiego et al., 2019; Zeng et al., 2022). Activating transcription factor 3 (ATF3) plays a vital role in modulating immunity. When ATF3 is activated, it forms a complex with c-Jun. Subsequently, this complex attaches to the promoters of cytokine genes, such as IL-1β, thereby inducing heightened cytokine production (Ku and Cheng, 2020). LYZ encodes for human lysozymes. Furthermore, it exhibits antibacterial properties against various bacterial species, playing a key role in the innate cellular antiviral response (NCBI, 2024b). While the identified genes have all been previously reported in CKD cases, this serves to demonstrate the efficacy of machine learning in pinpointing critical genes.

A significant feature of our study is the unique integration of Lasso and RF methods, which resulted in remarkable predictive performance. The feature selection method of Lasso (Ghosh and Chinnaiyan, 2005; Tsagris et al., 2018) and RF (C et al., 2023; Toth et al., 2019) has become a prevalent approach in biology for more effectively identifying essential biomarkers. Until now, no research has created a CKD prediction model utilizing gene sequencing, particularly due to the scarcity of kidney tissue samples from CKD patients, which are challenging to obtain.

Our model exhibited impressive AUC values of 1, 0.990, and 0.913 for the training dataset, validation dataset, and external validation dataset (GSE180394), respectively, within the context of array expression data. These results underscore the robustness of our model. Additionally, in the external validation dataset (GSE180394), our model displayed a commendable CKD sensitivity of 0.889. We investigated the accuracy and reliability of machine learning in predicting CKD risk at the gene transcriptome level. Consequently, we have successfully crafted an innovative CKD disease risk prediction model that can serve as a valuable tool for CKD risk assessment and disease identification.

Nonetheless, our study does have certain limitations: 1) Some of the publicly available datasets lack detailed clinical information about patients and control samples, limiting our ability to consider distinct CKD stages and additional comorbidities comprehensively. 2) Despite our efforts to merge multiple datasets to create a larger dataset for model building, the number of samples available for machine learning still falls short of ideal. Future work could involve including more research data in the training dataset to enhance model performance. 3) Addressing the challenge of model overfitting is a complex task, and while we employed a 10-fold cross-validation approach during model construction to mitigate overfitting, it may not completely eliminate the problem. Real-world data often contains noise, and the model’s generalization ability may not be as strong as indicated by validation results. 4) The model has yet to undergo testing in practical applications for predicting CKD patients. Therefore, additional research data will be essential in the future to assess the model’s robustness and its ability to generalize effectively.

## Conclusion

In summary, our comprehensive analysis of the CKD dataset from the GEO database revealed that the critical biomarkers DUSP1, GADD45B, IFI30, IFI44L, ATF3, and LYZ, which exhibited significant associations with CKD, collectively formed a robust disease risk prediction model for CKD using the random forest algorithm. Notably, this study marks the first use of random forest machine learning techniques to develop a robust predictive model for CKD based on these six genes.

## Data Availability

The original contributions presented in the study are included in the article/Supplementary Material, further inquiries can be directed to the corresponding author.

## Supplementary material

SUPPLEMENTARY FIGURE S1 Box plot illustrating gene distribution pre- and post-batch effect adjustment, with batch correction applied to datasets GSE35488, GSE32591, GSE66494, and GSE47184.

SUPPLEMENTARY FIGURE S2 Box plot depicting the distribution of genes prior to and following the correction of batch effects. This adjustment was made on the combined dataset, encompassing the training and validation datasets, along with the GSE180394 dataset.

SUPPLEMENTARY FIGURE S3 Receiver Operating Characteristic (ROC) curves comparing the performance of the model with OAS3 and the model excluding OAS3 in the validation dataset.

SUPPLEMENTARY TABLE S1 The information on the platform and research topics on GEO’s Homo Sapien CKD datasets.
