# Supplementary figures and images for: Development and evaluation of a chronic kidney disease risk prediction model using random forest
Source: Front Genet. 2024 Jun 27;15:1409755. doi: 10.3389/fgene.2024.1409755 (PMC11236722; doi:10.3389/fgene.2024.1409755)

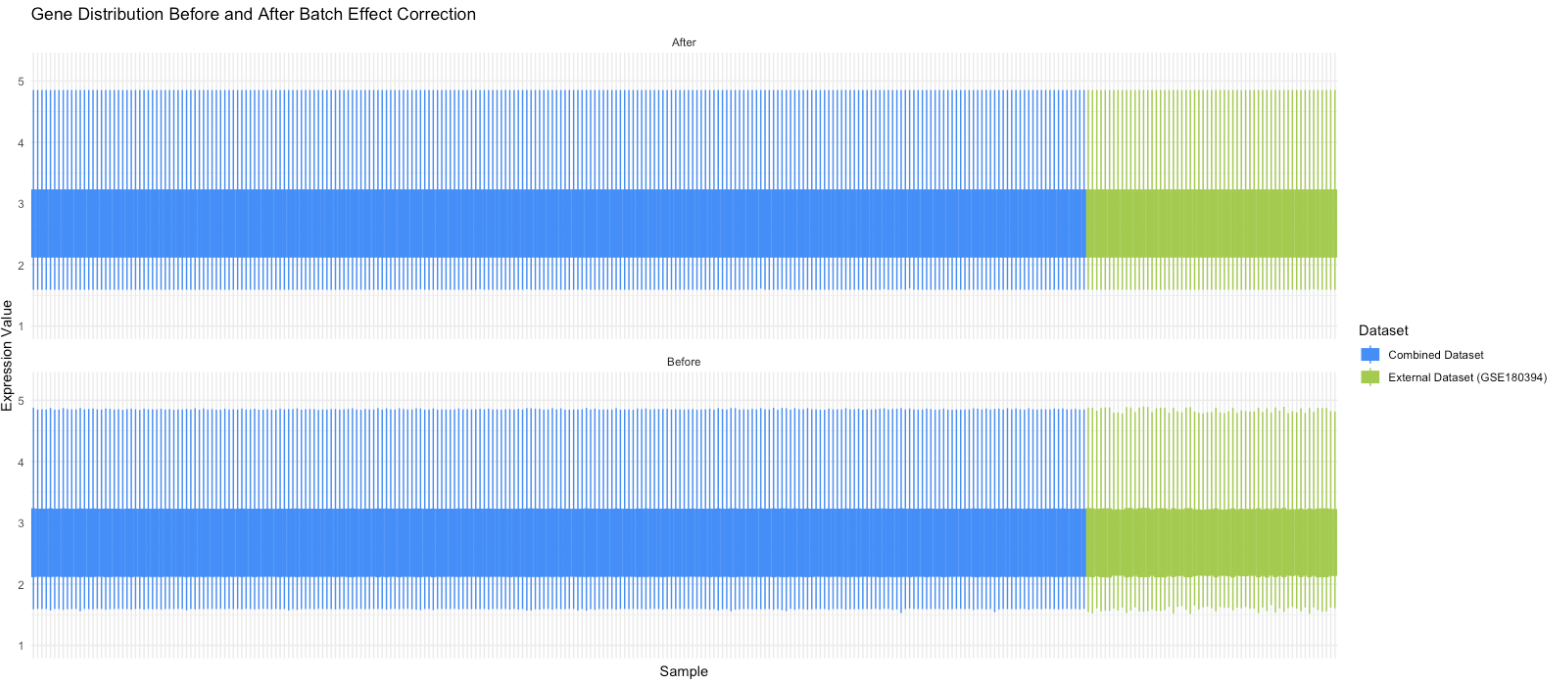

Supplement: Supplementary file 1 [file Image2.png]

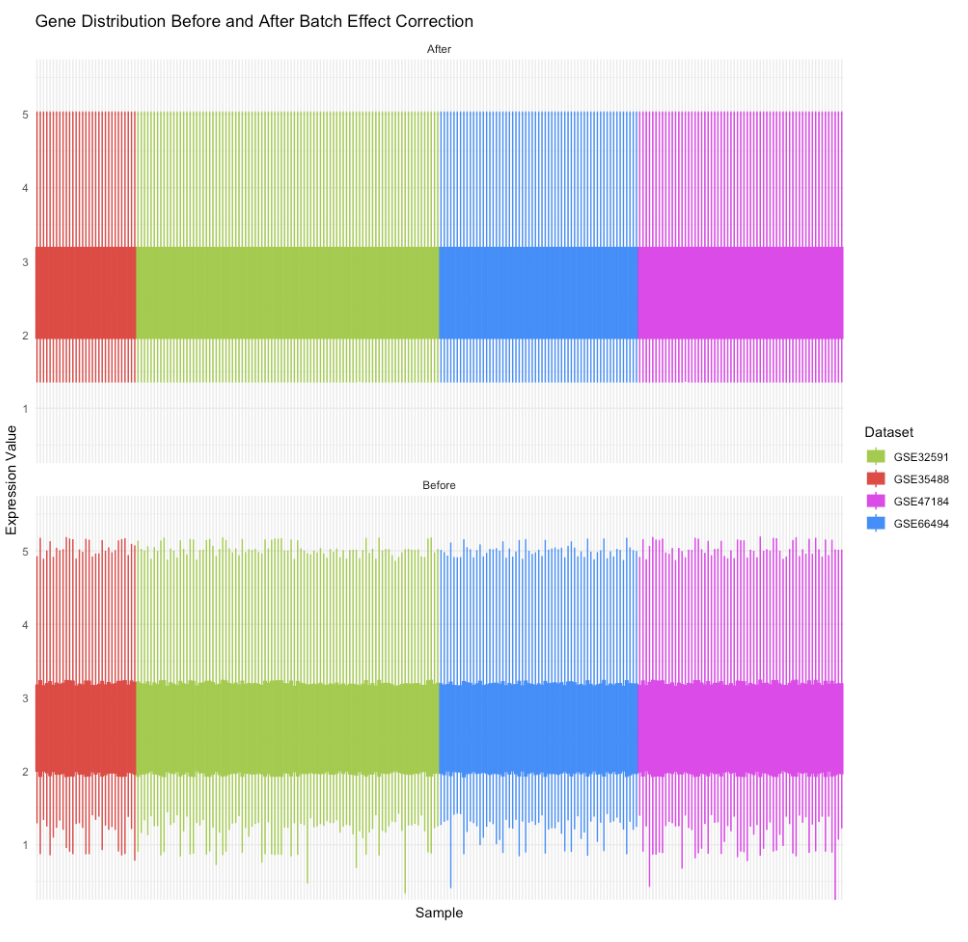

Supplement: Supplementary file 3 [file Image1.png]

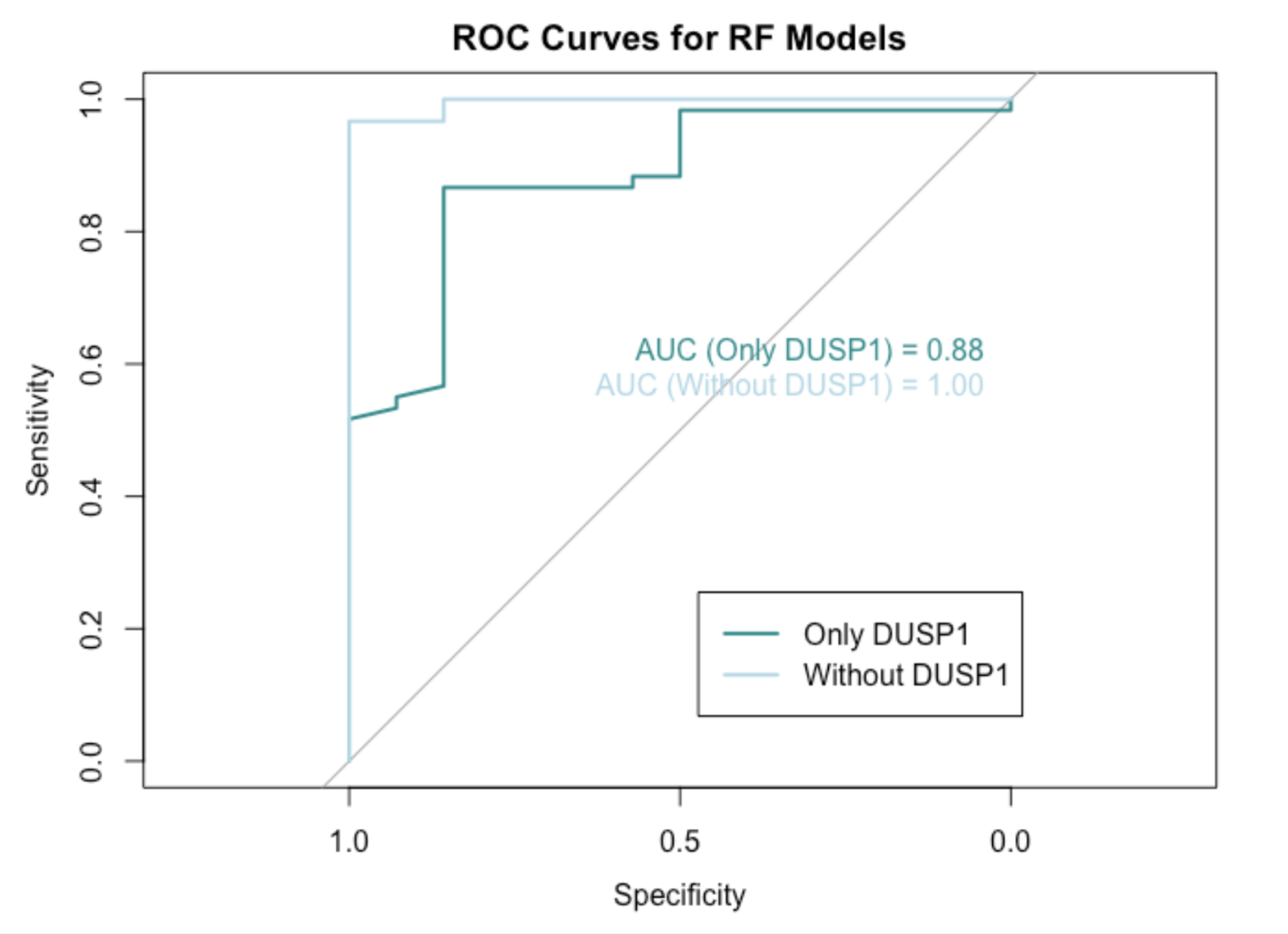

Supplement: Supplementary file 4 [file Image3.png]
